# Profile of the Spleen Transcriptome in Beef Steers with Variation in Gain and Feed Intake

**DOI:** 10.3389/fgene.2016.00127

**Published:** 2016-07-25

**Authors:** Amanda K. Lindholm-Perry, Rebecca J. Kern, Brittney N. Keel, Warren M. Snelling, Larry A. Kuehn, Harvey C. Freetly

**Affiliations:** U.S. Meat Animal Research Center, Agricultural Research Service, United States Department of AgricultureClay Center, NE, USA

**Keywords:** beef cattle, copy number variation, feed intake, gain, spleen, transcriptome

## Abstract

We have previously identified components of the immune system contributing to feed intake and gain in both the rumen and small intestine of beef steers. In this study, we examined the spleen, a major lymphatic organ near the digestive tract, to determine whether it was also influencing individual feed efficiency status through immune responses. Animals (*n* = 16) that were divergent for gain and intake were selected for tissue sampling. The spleen transcriptomes were evaluated by microarray. A total of 1216 genes were identified as differentially expressed. Genes were over-represented in Kyoto encyclopedia of genes and genomes (KEGG) pathways including biological regulation, protein folding, cell communication, immune systems process, response to stress, and RNA metabolic process. Several stress response or heat shock genes including *HSPH1, HSPA1A, HSPA4, DNAJB4, DNAJA4*, etc., were identified as a stress response functional gene cluster in the low gain-low intake animals. These genes were up-regulated amongst the low gain-low intake animals compared to all other groups. Canonical pathways associated with the differentially expressed genes included the coagulation system, extrinsic prothrombin activation, protein ubiquitination, unfolded protein response, and aldosterone signaling in epithelial cells. An analysis of expressed copy number variable (CNV) genes in the spleen produced some of the same genes and gene families that were differentially expressed. Our data suggests the splenic contribution to some of the underlying variation among gain and intake within this group of animals may be a result of immune function and stress response. In addition, some of the differences in immune response functions may be related to gene copy number.

## Introduction

The spleen is responsible for filtering the blood of damaged red cells and microbes, and also serves as an immune organ. The spleen's roles in immune functions include surveying the blood for antigens and producing antibodies. The spleen is composed of red and white pulp. The red pulp contains platelets and phagocytes, the latter of which engulf the red blood cells, and pathogens that are filtered out of the blood stream. The phagocytes and platelets are also available for release into the blood stream when necessary to facilitate wound healing. The white pulp contains T and B cells, and macrophages and responds to the antigens circulating through the bloodstream.

Previous studies have examined spleen size for a relationship with feed efficiency in livestock. Meyer et al. (2015) found that spleen mass tended to be larger in sheep with lower residual feed intake regardless of diet type. In an unrelated study, Gain:Feed was also positively correlated with spleen size in beef cattle (Mader et al., 2009). It is possible that the size of the spleen may increase in response to infection, and perhaps the more feed efficient animals are responding to exposure to pathogens in a manner that is also favorable for feed efficiency.

Previously, we identified immune and inflammation components among differentially expressed genes associated with variation in feed intake and gain phenotypes in the rumen and small intestine transcriptomes of beef cattle (Kern et al., 2016; Lindholm-Perry et al., 2016). For example, the antigen processing/presentation and pathways involved in the pathogenesis of influenza were identified in the small intestine of high gain vs. low gain animals. In the rumen, pathways including interferon signaling and PI3K signaling in B lymphocytes were over-represented in high vs. low gaining animals. It is possible that some of the immune responsiveness of these animals to routine exposure to pathogens is a result of splenic activity as a response to circulating blood antigens that are filtered through the spleen. In this study, we have investigated beef steers for variation in their spleen transcriptomes.

## Materials and methods

### Animal care and use

The U.S. Meat Animal Research Center (USMARC) Animal Care and Use Committee reviewed and approved all animal procedures. The procedures for handling cattle complied with the Guide for the Care and Use of Agricultural Animals in Agricultural Research and Teaching (FASS, 2010).

### Population

Steers selected for this study were born in the fall of 2012 and originated from the continuous phase of the USMARC Germplasm Evaluation project (Schiermiester et al., 2015; Table 1), a breeding program intended to develop a population of cattle with high percentages of the following breeds: Angus, Beefmaster, Brahman, Brangus, Braunvieh, Charolais, Chiangus, Gelbvieh, Hereford, Limousin, Maine Anjou, Red Angus, Salers, Santa Gertrudis, Shorthorn, and Simmental. Each year the heifers and cows were artificially inseminated to prominent industry bulls of their dominant breed.

**Table 1:** **Breed composition of steers by phenotypic group**.

**Breed**	**High gain-high intake**	**High gain-low intake**	**Low gain-low intake**	**Low gain-high intake**	**High gain**	**Low gain**	**High intake**	**Low intake**
Hereford	0.125	0.3125	0.3125	0.0625	0.21875	0.1875	0.09375	0.3125
Angus	0.21875	0.25	0	0.1875	0.234375	0.09375	0.203125	0.125
Shorthorn	0	0.1875	0	0	0.09375	0	0	0.09375
Brahman	0	0	0.125	0	0	0.0625	0	0.0625
Simmental	0	0	0	0.25	0	0.125	0.125	0
Maine Anjou	0	0	0.1875	0	0	0.09375	0	0.09375
Gelbvieh	0	0	0	0.1875	0	0.09375	0.09375	0
Brangus	0.125	0	0	0	0.0625	0	0.0625	0
Santa Gertrudis	0.1875	0	0	0	0.09375	0	0.09375	0
MARCII^a^	0	0	0.0625	0	0	0.03125	0	0.03125
MARCIII^b^	0.125	0	0	0	0.0625	0	0.0625	0
Salers	0.1875	0.125	0	0	0.15625	0	0.09375	0.0625
Beefmaster	0	0	0	0.125	0	0.0625	0.0625	0
Chiangus	0	0.0625	0.1875	0.125	0.03125	0.15625	0.0625	0.125
Charolais	0.03125	0	0	0	0.015625	0	0.015625	0
SIMMENTAL × MARC I^c^	0	0.0625	0	0.0625	0.03125	0.03125	0.03125	0.03125
MARC II-CHAROLAIS-MARC III	0	0	0.0625	0	0	0.03125	0	0.03125
UNK^d^	0	0	0.0625	0	0	0.03125	0	0.03125

a *MARCII animals consist of the following breed composition: 1/4 Simmental, 1/4 Gelbvieh, 1/4 Hereford, 1/4 Angus*.

b *MARCIII animals consist of the following breed composition: 1/4 Pinzgauer, 1/4 Red Poll, 1/4 Hereford, 1/4 Angus*.

c *MARCI animals consist of the following breed composition: 1/4 Limousin, 1/4 Brown Swiss,1/4 Charolais,1/8 Hereford,1/8 Angus*.

d *UNK corresponds to unknown breed of origin*.

### Feed efficiency phenotypes

Steers (*n* = 148) were evaluated for feed intake using the Insentec feeding system (Marknesse, The Netherlands). The trial began when the steers were ~1 year of age and lasted for an 84-day period. Steers had *ad libitum* access to a ration (DM basis) consisting of: 57.35% dry-rolled corn, 30% wet distillers grains with solubles, 8% ground alfalfa hay, 4.25% vitamin, and mineral supplement containing Monensin (Land O'Lakes Feed LLC, Gray Summit, MO, USA), and 0.4% urea. Body weight was measured on days 0 and 1, 83 and 84, and at least every 3 weeks during the study. Body weight was regressed using a quadratic function on days on study, and total gain was calculated from the resulting regression equation. At the end of each feeding period, steers were ranked based on their standardized distance from the bivariate mean (ADG and ADFI) assuming a bivariate normal distribution with a calculated correlation between ADG and ADFI. Four steers with the greatest deviation within each Cartesian quadrant were sampled (*n* = 4/group). In the event that a sire breed was over-represented within a quadrant, a steer with the next highest rank of a different breed was selected. The result was a 2 × 2 factorial design consisting of greater and less ADFI, and greater and less ADG. Animals with medical or health issues (i.e., pneumonia or foot rot) that might affect gain or intake were excluded prior to the selection process. Breed composition by phenotypic group is presented in Table 1.

### Tissue collection and RNA isolation

Animals selected for this study were comingled in a pen with *ad libitum* access to the same diet. Animals were slaughtered over a consecutive 4-day period, with one animal from each of the phenotypic groups represented during each harvest day. The steers were able to consume feed and water until their weights were taken on the morning of slaughter and transported to the US Meat Animal Center abattoir (under 6.4 km). On each slaughter day, the four animals selected were processed serially within a 3-h time frame. The spleen tissue was obtained from each steer at ~30 min post-exsanguination. A portion of the spleen was removed from the unattached end of the spleen, ~8–10 cm from the free end of the spleen. Small sections of the spleen tissue were diced into 50–100 mg samples from middle of the open end of the spleen slice. These samples were immediately frozen in liquid nitrogen and stored at −80°C until they could be processed further. RNA was isolated with TriPure Reagent (Roche, Indianapolis, IN, USA) according to the manufacturer's protocol with an extra 20 min centrifugation following the addition of chloroform. The resulting RNA pellets were centrifuged through RNeasy Plus Mini Kit columns (Qiagen, Valencia, CA, USA) according to the manufacturer's instructions to remove genomic DNA.

### Microarray analysis

Each sample (1 uL) was evaluated using the Nanodrop 8000 (Thermo Scientific, Wilmington, DE, USA) for OD and A260/280 ratios. A260/280 ratios were above 1.8 and concentrations were above 85 ng/uL for all samples tested. In addition, all samples were evaluated for integrity using the Agilent Bioanalyzer 2100 with RNA Nano 6000 chips. Samples with RIN values of RIN ≥ 5.8 were processed. The average RIN values for all 16 samples was 8.0.

To assess the differential expression of the genes in the spleen of steers, the Affymetrix GeneAtlas System (Santa Clara, CA) in conjunction with Bovine 1.1ST array strips were used according to the manufacturer's protocol. Briefly, 250 ng of total RNA and *Poly-A* RNA controls were converted to single stranded cDNA, then fragmented and TdT labeled with the WT Expression Kit. A hybridization cocktail was prepared from fragmented cDNA, *bioB, bioC, bioD*, and *cre* controls, and Control Oligonucleotide B2 using the Affymetrix GeneAtlas Hybridization, Wash and Stain Kit for WT Array Strips, and GeneChip Hybridization Controls (Santa Clara, CA). Arrays were hybridized for 20 h at 48°C. Staining and washing was completed utilizing the GeneAtlas Fluidics station. Chips were immediately imaged in a calibrated Affymetrix GeneAtlas scanner, and all arrays passed the imaging quality control assessment. CEL formatted files were annotated using Affymetrix Expression Console (Colombo et al., 2014). Guanine Cytosine Count Normalization (GCCN) and Signal Space Transformation (SST) algorithms were applied to the microarray data. The GCCN program normalizes the signal of the probes by GC content. The SST algorithm was used to stretch the signal intensity distribution in order to decompress the fold change ratios. Samples were then subjected to Robust Multichip Analysis (RMA) for processing, further normalization and analysis of the microarrays (Irizarry et al., 2003). The CEL and CHP files have been submitted to the NCBI Gene Expression Omnibus (GEO) database (Accession Series GSE73261) for possible inclusion in meta-analyses. Expression pattern differences were analyzed using a model with Cartesian quadrant (high intake-high gain; low intake-high gain; low intake-low gain; high intake-low gain) as a fixed effect and animal as residual with SAS, Inc. (Cary, NC, USA). Least squares mean were derived for genes with significant expression patterns in a three degree of freedom test. Significance values were multiple-test corrected for 24,431 genes using the FDR procedure of Benjamini and Hochberg (1995). *P*-values of contrasts are also reported for gain (high gain quadrants minus low gain quadrants), intake (high intake quadrants minus low intake quadrants), and their interaction.

### Validation by qRT-PCR

Genes (*n* = 13) were chosen to validate the microarray data using real-time PCR. Complimentary DNA (cDNA) from the RNA isolated from the spleen from the same 16 animals sampled for microarray was used to perform real-time PCR. Primer 3 was used to design primer sets (http://primer3.ut.ee/). Primers for 18S were tested across all animals. No statistically significant differences between groups were detected (*P* > 0.2). Real-time PCR was performed in duplicate for each gene and triplicate for the 18S housekeeping gene and pooled samples using the Bio-Rad CFX384 instrument (Bio-Rad, Hercules, CA, USA) and SYBR Green master mix (Bio-Rad) with 5 ng of cDNA template. The real-time PCR reaction was performed at 95°C for 5 min followed by 45 cycles of 95°C for 10 s, 55°C for 10 s, and a final melting curve from 65 to 95°C. The Bio-Rad CFX384 software calculated the threshold cycle (*Cq*) for each gene from each sample with 18S as the reference gene. Analysis of qRT-PCR data was performed using the *Cq* of the target gene and the reference gene to calculate the Δ*C*_t_. The *Cq*-values from each sample and a pooled control sample were used to calculate the ΔΔ*C*_t_. Fold changes between samples were determined using log transformed 2^−ΔΔ*C*t^ obtained using the Livak and Schmittgen (2001) method.

### Biological process analysis

The Protein Analysis Through Evolutionary Relationships (PANTHER) classification system (pantherdb.org) was chosen to evaluate the list of spleen DEG for enrichment among biological processes. The list of 1137 annotated genes was evaluated using the statistical overrepresentation test with the *B. taurus* database and the default parameters. Processes were considered significant at *P* < 0.05.

### Qiagen ingenuity pathway analysis

Ingenuity® Pathway Analysis (IPA®, QIAGEN) was used to evaluate the list of DEG for over-representation in canonical pathways. The software depicted systems-level gene interactions, based on the Ingenuity Knowledge Base database. Data were imported in a flexible format using gene symbol as the identifier, and genes with *P* < 0.05 were used for core analyses. Top networks were identified for all DEG genes. The program calculates a *P*-value for each network using the fit of the significant genes and the size of the network.

### Copy number variable (CNV) gene discovery

Whole exome sequencing was performed on a separate population of 175 bulls used in Cycle VII of the USMARC Germplasm Evaluation project (Snelling et al., 2015). This set of animals included 122 purebred AI sires representing 10 different breeds, and 53 F_1_ natural service sires representing 10 different crosses of 7 breeds. Exome sequencing of the bulls was previously described by Snelling et al. (2015). Genomic DNA was extracted from semen and blood using standard DNA extraction protocols (phenol-choloroform extraction for semen and QIAamp DNA Mini Kit for blood), and sheared to an average size of 300 bp. Indexing adapters were added to allow identification of individual DNA samples from pools of 8 samples. The Agilent SureSelect Target Enrichment System Kit I and Kit II (Agilent Technologies, Inc., Santa Clara, CA) were used to generate a DNA library for each sample. Equal quantities of each indexed DNA library were pooled into groups of 8 for exome capture using the Agilent SureSelect XT Bovine capture reagent (Agilent Technologies, Inc., Santa Clara, CA). Exome capture libraries were then sequenced with the Illumina MiSeq technology (MiSeq Reagent Kit V2 and V3 chemistry; Illumina, San Diego, CA) to obtain a mean 20x coverage of targeted intervals.

Copy number variation was detected in the exome sequence. The cn.MOPS algorithm (Klambauer et al., 2012) was used to identify putative CNV. cn.MOPs is a multiple sample read depth CNV detection method that applies a Bayesian approach to decompose read variations across multiple samples into integer copy numbers and noise by its mixture components and Poisson distributions, respectively. cn.MOPS avoids read count biases along the chromosomes by modeling the depth of coverage across all samples at each genomic position. The exome version of the cn.MOPS program was run using the default parameters.

The resulting CNVs were then used to construct a set of copy number variable regions (CNVRs). A CNVR was constructed by merging CNVs within and across samples that exhibited at least 50% pairwise reciprocal overlap in their genomic coordinates. This method identified 131 CNVRs (119 on the autosomes and 12 on the X chromosome). Sizes of the CNVRs ranged from 0.0016 to 1.56 Mb, with an average of 0.1097 Mb, and a median of 0.0369 Mb. The CNVRs occupied a total of 12.0038 unique Mb or 0.45% of the UMD 3.1 *Bos taurus* genome.

Genes from the RefSeq annotation of UMD 3.1 overlapping (both completely and partially) with CNVRs were then identified. A total of 353 protein-coding genes were overlapped by CNVRs.

### Expressed CNV genes in the spleen

To identify expressed CNV genes in the spleen, the microarray data was processed as follows. Individual CEL files were processed using the UPC function from the SCAN.UPC package in R (Piccolo et al., 2012, 2013). UPC is a quantitative approach for normalizing gene expression data that produces standardized expression values that estimate whether a gene is “active” in a given sample. The program outputs for each gene in a given sample a universal expression code (UPC), a number between 0 and 1 where larger values suggest a greater likelihood that the gene is expressed in the sample. The UPC function was run using the default parameters, and a gene was considered to be expressed in the spleen if it had a UPC > 0.3 in at least one sample. The set of CNV genes expressed in the spleen was then identified by finding the overlap between the set of genes expressed in the spleen and the set of CNV genes. The CNV genes were analyzed using the PANTHER classification system for genes to obtain over-represented biological processes. Significance of gene ontology (GO) terms was assessed using the default *B. taurus* GO annotation as background for the enrichment analysis, and terms were considered significant at a Bonferroni corrected *P* < 0.05.

## Results

### Phenotypic measurements

Table 2 lists the gain and feed intake means, minimums and maximums for all four phenotypic groups of animals (*n* = 4/group). Briefly, the high gain-high intake animals gained on average 187 kg and consumed 1210 kg of dry matter over the course of the 84-day trial. The high gain-low intake animals gained an average of 166 kg and consumed 762 kg of dry matter over the study. The low gain-low intake animals gained 135 kg and consumed 743 kg of dry matter over the trial; and the low gain-high intake group gained 124 kg on 1135 kg of feed. Averages for the animals grouped as high and low gain, and high and low intake are also included (Table 2).

**Table 2:** **Average, minimum, and maximum values for the total gain and feed intake for 16 steers (*n* = 4/group) over 84 days on study**.

**Group^a^**	**Gain (kg)^b^**	***SD***	**Feed intake (kg)^c^**	***SD***
High gain-high intake	187	18.2	1210	202.5
High gain-low intake	166	6.8	763	93.4
Low gain-low intake	135	23.8	743	33.8
Low gain-high intake	124	28.5	1135	126.4
High gain	176	16.8		
Low gain	130	25.0		
High intake			1172	161.3
Low intake			753	65.9

a *Animal groups (n = 4 animals) in each group of high gain-high intake, high gain-low intake, low gain-low intake, and low gain-high intake. High gain and low gain groups, as well as high intake and low intake groups are composed of 8 animals*.

b *Gain is expressed in total kg in body weight gained over the trial*.

c *Feed intake is expressed in total kg of dry matter intake consumed during the feeding trial*.

### Differential gene expression

Analysis of the microarray data produced 1216 differentially expressed genes (DEG) with nominal *P*-values of < 0.05. Thirty of these genes (27 annotated genes) displayed *P* < 0.001. These included a heat shock gene (*HSPH1*); a transcription factor (*TSHZ2*); and two ion channels (*KCNMA1* and *SCN4B*). This gene list also includes two genes that appear to be involved in immune function: *ADORA2A*, which affects spleen size and number of naïve T-cells in mice (Cekic et al., 2013) and *SAA4*, a major acute phase reactant. *HADHB* was also identified and this gene encodes a mitochondrial enzyme that catalyzes the final steps of beta-oxidation of long chain fatty acids. The lowest FDR value for these genes was 0.75. The complete list of DEG with *P*-values, fold change, and FDR data is presented in Supplemental Table 1.

Contrasts for gain, intake, and their interaction were also derived. From the genes nominally significant for quadrant differences, 654 genes were differentially expressed (*P* < 0.05) for gain; 404 for intake, and the 494 genes for their interaction (Supplemental Table 1).

### Validation with qRT-PCR

Differentially expressed genes (*n* = 13) were evaluated using qRT-PCR to validate the microarray data. Fold change expression data for microarray and qRT-PCR for each gene are presented in Table 3. Six genes were associated with phenotypic group; however, one of these displayed the opposite direction of the microarray data (*P* < 0.1). Seven of the genes were in high concordance with the microarray data (*r* > 0.85) and two more were in concordance with lower correlation (*r* > 0.5). The three genes with the most significant differences also had correlations between microarray and qRT-PCR of *R* ≥ 0.95.

**Table 3:** **qRT-PCR validation of genes (*n* = 13) identified by microarray analysis as differentially expressed in the spleen of beef steers with variation for feed intake and weight gain**.

	***PPAP2C***		***CREM***		***DNAJC5***		***BAG3***		***DNAJB1***	
	**qRT-PCR^a^**	**Microarray^b^**	**qRT-PCR**	**Microarray**	qRT-PCR^*^^c^	**Microarray**	qRT-PCR^**^^d^	**Microarray**	qRT-PCR^**^	**Microarray**
High gain-high intake	0.064	7.392	0.09	8.565	−0.0515	10.624	−0.145	9.749	0.524	12.184
High gain-low intake	−0.205	7.331	−0.0991	8.258	−0.0371	10.609	−0.312	9.474	0.563	12.210
Low gain-low intake	−0.331	7.106	−0.0142	9.270	0.0322	9.999	0.338	10.929	0.975	14.027
Low gain-high intake	−0.209	6.974	−0.0238	8.379	0.0441	10.097	−0.196	9.237	0.525	12.255
*SE*^e^	0.0152	0.0708	0.097	0.106	0.028	0.172	0.102	0.294	0.082	0.430
	***HADHB***		***SCN4B***		***GBP7***		***HSPH1***			
	**qRT-PCR**	**Microarray**	qRT-PCR^*^	**Microarray**	qRT-PCR^*^	**Microarray**	qRT-PCR^**^	**Microarray**		
High gain-high intake	−0.0211	11.741	−0.336	7.712	−0.102	9.982	−0.0486	12.823		
High gain-low intake	−0.055	11.333	−0.378	7.398	0.0171	11.177	−0.172	12.443		
Low gain-low intake	−0.0841	11.329	0.127	7.946	0.0443	10.324	0.409	15.450		
Low gain-high intake	−0.0789	11.371	−0.173	7.638	−0.00681	10.111	−0.175	12.164		
*SE*	0.053	0.0541	0.134	0.0613	0.0707	0.235	0.117	0.448		
	***KCNMA1***		***LPIN1***		***SIRT6***		***TSHZ2***			
	**qRT-PCR**	**Microarray**	**qRT-PCR**	**Microarray**	**qRT-PCR**	**Microarray**	**qRT-PCR**	**Microarray**		
High gain-high intake	0.15	7.806	−0.0992	9.393	0.11	9.313	0.102	9.651		
High gain-low intake	−0.0231	7.218	−0.118	9.310	0.0874	9.593	−0.0294	9.347		
Low gain-low intake	−0.0193	7.553	−0.121	10.114	0.0579	9.250	−0.0895	8.737		
Low gain-high intake	0.23	8.201	−0.0345	9.404	0.0931	9.500	0.0511	9.733		
*SE*	0.129	0.108	0.133	0.167	0.0862	0.0567	0.0673	0.117		

a *Log transformed 2^−ddCT^ values from qRT-PCR*.

b *Relative expression from microarray data log 2 transformed*.

c ^*^P ≤ 0.01.

d ^**^P ≤ 0.05.

e *Standard errors given for qRT-PCR and microarray analyses*.

### Gene ontology and pathway over-representation among DEG

The Panther statistical overrepresentation test for biological processes produced the following categories: Biological regulation, protein folding, cell communication, and RNA metabolic process (Table 4). The IPA® software was used to determine which pathways were over-represented by the genes identified as DEG. The canonical pathways identified included unfolded protein response, protein ubiquitination, coagulation system, aldosterone signaling in epithelia cells, and endoplasmic reticulum stress pathway (Table 5).

**Table 4:** **Functional gene clusters represented in the list of differentially expressed genes in the spleen of beef steers with gain and feed intake variation**.

**PANTHER GO-slim biological process^a^**	***B. taurus* ref^b^**	**Number of DEG^c^**	**Expected^d^**	**Fold enrichment^e^**	***P*-value**
Biological regulation	3868	169	141.9	1.19	7.27E-03
Regulation of biological process	2803	126	102.83	1.23	9.21E-03
Death	595	32	21.83	1.47	2.24E-02
Cell death	591	32	21.68	1.48	2.07E-02
Apoptotic process	577	32	21.17	1.51	1.54E-02
Negative regulation of apoptotic process	93	10	3.41	2.93	2.70E-03
Protein folding	154	14	5.65	2.48	2.05E-03
Cell communication	3113	134	114.2	1.17	2.64E-02
Immune system process	1477	68	54.18	1.25	3.32E-02
Neurological system process	1234	57	45.27	1.26	4.60E-02
Regulation of transcription from RNA polymerase II promoter	1137	53	41.71	1.27	4.65E-02
Response to stress	704	39	25.83	1.51	8.18E-03
RNA metabolic process	2109	92	77.37	1.19	4.72E-02
RNA splicing	137	11	5.03	2.19	1.39E-02
RNA splicing, via transesterification reactions	134	10	4.92	2.03	2.84E-02
Unclassified	7560	238	277.34	0.86	1.40E-03
Cellular component morphogenesis	462	10	16.95	0.59	4.84E-02
DNA replication	167	1	6.13	< 0.2	1.53E-02

a *Biological process enriched for in the list of DEG genes*.

b *Number of genes in the bos taurus genome that are included in the biological process*.

c *Number of DEG identified within the biological process*.

d *This is the number of genes that were expected based on the reference genes*.

e *Fold Enrichment of the genes observed in the list of DEG genes that is over the expected*.

**Table 5:** **Canonical pathways identified by Ingenuity pathway analysis from the genes differentially expressed in the spleen of beef steers with variation in feed intake and gain**.

**Name^a^**	***P***	**Gene symbols^b^**	**Number of DEG^c^**	**No. genes in pathway^d^**
Coagulation system	0.005	BDKRB2, F2, F3, F5, PLAUR, SERPINC1	6	33
Extrinsic prothrombin activation pathway	0.005	F2, F3, F5, SERPINC1	4	15
Protein ubiquitination pathway	0.005	DNAJA1, DNAJB1, DNAJB4, DNAJC5, DNAJC16, HSCB, HSP90AA1, HSPA4, HSPA6, HSPA8, HSPH1, PSMB10, PSMC5, PSMD7, SKP1, SUGT1, TRAF6, UBE2D3, UBE2N, UBE2Q1, USP30	21	235
Unfolded protein response	0.01	CEBPA, DDIT3, HSPA4, HSPA6, HSPA8, HSPH1, INSIG1	7	50
Aldosterone signaling in epithelial cells	0.02	DNAJA1, DNAJB1, DNAJB4, DNAJC5, DNAJC16, HSCB, HSP90AA1, HSPA4, HSPA6, HSPA8, HSPH1, PLCD1, SCNN1A	13	143

a *Canonical pathways over-represented from the list of 1,216 DEG identified in the spleen*.

b *List of genes represented in each pathway listed in HUGO gene symbol format*.

c *The number of genes from list of DEG from the spleen that are represented in each pathway*.

d *The total number of genes represented in each pathway*.

**Table 6:** **Biological processes and GO annotation identified as over-represented among copy number variation genes identified in the spleen of beef cattle with variation in feed intake and gain**.

**Biological process^a^**	**GO number^b^**	**Bos taurus ref^c^**	**Number of CNV genes**	**Expected^d^**	**Fold enrichment^e^**	***P*^f^**	**Genes^g^**
Response to interferon-gamma	GO:0034341	44	4	0.1	>5	0.0008	GBP2, GBP4, LOC781675, LOC781710
Antigen processing and presentation of peptide or polysaccharide antigen via mhc class ii	GO:0002504	36	3	0.08	>5	0.02	BOLA-DQB, BOLA-DQA2, BOLA-DQA5
Macrophage activation	GO:0042116	164	5	0.38	>5	0.009	DMBT1, GBP2, GBP4, LOC781675, LOC781710
Immune system process	GO:0002376	1477	12	3.42	3.8	0.005	BOLA-DQB, GIMAP4, GIMAP8, GIMAP7, LOC511617, DMBT1, BOLA-DQA2, BOLA-DQA5, GBP2, GBP4, LOC781675, LOC781710

a *Biological process enriched for in the list of CNV genes*.

b *Gene ontology annotation identification number*.

c *Number of genes in the bos taurus genome that are included in the biological process*.

d *This is the number of genes that are expected based on the reference genes*.

e *Fold Enrichment of the genes observed in the list of CNV genes compared to the number expected*.

f *Bonferroni corrected P-value*.

g *HUGO gene symbol representing CNV genes expressed in the spleen that are over-represented in each of the biological process categories*.

No genes were over-represented for biological processes for gain or intake; however, protein folding was over-represented among the genes differentially expressed the interaction of gain and intake (Bonferroni corrected *P* = 9.6 × 10^−4^). The genes included *BAG3, HSP90AA1, FKBP4, HSPA4, HSPA6, STIP1, DNAJB4, AHSA1, SUGT1, AHSA2, HSPA8*, and *HSPH1*.

### Copy number variation

A total of 60 CNV genes were identified as expressed in the spleen of the steers used in this study. GO enrichment analysis of these genes was conducted using PANTHER's statistical over-representation test with Bonferroni correction for multiple testing. Four biological process terms were over-represented in the expressed splenic CNV genes (Table 6). These were response to interferon-gamma, antigen processing, and presentation of peptide or polysaccharide antigen via MHC class II, macrophage activation, and immune system process. One of the genes associated with the antigen processing/presentation biological process was BOLA-DQB. This gene was identified as both a CNV gene and a DEG. The CNV genes GBP2, GBP4, and LOC781675 (or GBP7) were associated with the other three biological process terms. The GBP7 gene and the related family member gene, GBP6, were identified as DEG by microarray. The CNV region encompassing GBP2, GBP4, and GBP7 was located at Chr3:54364565-54608154, and GBP6 is located slightly 3′ to the same CNV region.

## Discussion

The spleen is a major reservoir for immune cells and may be important for cattle that are routinely exposed to normal, environmental pathogens, especially in a feedlot setting where animals are in close proximity to each other. We previously identified immune and inflammatory response genes associated with specific feed intake and gain phenotypes in the rumen and small intestine. As a result, the purpose of this study was to evaluate the spleen in cattle with divergent feed intake and gain to determine whether DEG in this tissue might also have a role in these phenotypes.

We chose to use crossbred cattle for our study in order to detect DEG that are robust and involved in feed intake and gain across multiple breeds and populations of cattle. The cattle in this population include 16 breeds and 4 animals per phenotypic group were selected, thus it was not possible to attain equivalent breed diversity across all groups of cattle in order to select those animals with the most divergent phenotypes. We have attempted to address breed composition within each group of animals by ascertaining that breeds were not over-represented. Many of the breeds were represented in more than one of the phenotyping allocations sampled or they were represented in relatively low percentages of composition to help reduce hidden breed effects.

By chance, we expected to identify 1220 genes (α = 0.05 and 24,431 genes). Our analysis produced 1217 differentially expressed genes from the four phenotypic groups, of which none passed FDR, and suggests that our DEG were based on chance alone. To evaluate this further, we tested the expression of 13 genes using qRT-PCR. Nine of the 13 genes chosen for qRT-PCR produced the similar patterns of expression among the 4 phenotypic groups of cattle and the expression of six genes were significantly associated with phenotype at *P* < 0.1 by qRT-PCR. Then we examined the list of DEG for overrepresentation or enrichment using gene function and pathway analyses. Some of the DEG were grouped into pathways and functional gene clusters providing additional support for their potential role in cattle production traits. A CNV analysis of genes expressed in the spleen also produced some overlap with the DEG identified in this study. Moreover, some of the genes identified as differentially expressed in the spleen of the animals in this study were also identified in the rumen of steers with the similar gain and intake phenotypes, but collected during a different year (Kern et al., 2016) and/or the sections of small intestine of these animals (Lindholm-Perry et al., 2016). For example, a major histocompatibility class II gene (BOLA-DQB) was more highly expressed the low intake animals in the small intestine and the spleen tissues.

Immune response and/or antigen processing/presentation pathways were over-represented in almost every analysis that we performed, including the CNV analysis. Antigen processing/presenting was over-represented, in part, due to the expression of the BOLA-DQB gene which was more highly expressed in the low intake animals. The expression of major histocompatibility (MHC) class II genes BOLA-DQB may be regulated in part by cytokines (Boss, 1997). MHC class II present peptide antigens to T-cells to illicit immune responses and these genes are more highly expressed in the high gain-low intake animals than in the high gain-high intake and low gain-high intake LH animals. This same pattern of expression was detected in these animals in the small intestine suggesting that the increase in the expression of these immune genes may contribute to a more favorable feed utilization phenotype.

Response to interferon gamma and immune system response were also both identified by the CNV analysis. One CNV region spans the genes GBP2, GBP4, and GBP7. The genes GBP6 and GBP7 were both identified as differentially expressed in this study. The guanylate binding protein (GBP) family has been shown to respond to interferon gamma (Vestal and Jeyaratnam, 2011). These proteins may be important for resistance to pathogens. Degrandi et al. (2007) identified murine GBP1, 2, 3, 6, 7, and 9 expressed around the parasitophorous vacuole generated by the invasion of cells by *Toxoplasma gondii*. Another member of the GBP family of proteins, GBP1 appears to be involved in antiviral activities and GBP1 and 2 inhibit the growth of Chlamydia (Vestal and Jeyaratnam, 2011); thus, it is possible that GBP6 and 7 may have related antiviral or microbial inhibition functions. GBP6 was expressed at higher levels in the high gain compared to the low gaining animals in this study. The GBP7 gene was more highly expressed in the high gain-low intake animals compared to the other three groups (Supplemental Table 1). These functions and dosages of these genes may allow cattle to better respond to normal exposure to pathogens, either more quickly, or more efficiently, which in turn allows them to utilize energy to promote growth rather than to mount more energetically expensive immune responses.

We have identified a strong stress gene response in the spleen of the low gain-low intake animals compared to all other groups. In addition, the biological process protein folding was also identified as over-represented for gain by intake interactions, due to the expression of many of the same stress response genes. The genes HSP1A1, HSPA5 (Hsp70 family members), HSPH1 (an Hsp105kDa/110kDa protein 1 gene), and several DnaJ, also known as Hsp40 homolog genes, were up-regulated in the LL animals. This was also a pattern detected in these same animals along the small intestine (Lindholm-Perry et al., 2016). These genes tend to become over-expressed after exposure to stress, which may include extreme temperature, infection, inflammation, or oxidative stress (Santoro, 2000). With the up-regulation of multiple heat shock proteins within the LL group of animals, it seems that these steers are either experiencing or possibly reacting to some form of stress differently than the other groups. This evidence may also support the theory that animals in different gain and intake categories may have higher oxidative stress, which has been shown as a potential factor affecting feed efficiency in swine and cattle (Chen et al., 2011; Grubbs et al., 2013; Tizioto et al., 2015). Some types of Hsp genes within the spleen cells may be particularly reactive to stress events. Both cultured mouse spleen cells and the spleen cells of mice exposed to high temperature displayed increased levels of Hsp70 (Wieten et al., 2010), and both Hsp40 and Hsp70, among others, were increased in spleen of chickens exposed to arsenic (Guo et al., 2015). We cannot be sure what type of stressor event or whether the stress is internal (i.e., behavioral) or external, that might be affecting our population of animals, but it appears that the low gain-low intake animals have different biological stress responses than the other gain × intake groups that would have been exposed to the same external stress events.

Lipid metabolism was identified by IPA as an over-represented cellular or molecular function in DEG. Some of the DEG associated with this function were: *LPIN1, ELOVL5, PTGS2, SIRT6*, and *PPAP2C*. *Lipin 1* and *PPAP2C* are enzymes that function in the synthesis or concentration of long chain fatty acids and also in the triglyceride (TG) pathway. In humans and mice these genes are expressed in the spleen, and it may be of interest to evaluate them further. In humans, high triglyceride levels as a result of diabetes or obesity can cause splenomegaly, which can accompany weight loss. We detected LPIN1 as most highly expressed in the low gain-low intake animals and PPAP2C with decreased expression among the low gaining animals. While it is doubtful that our cattle are suffering from a disorder, it may be possible that, as in humans, regulation of triglyceride content is involved with gain.

Genes that are part of the aldosterone signaling pathway were identified by IPA as over-represented due to the heat shock genes and DNAJ genes up-regulated among the low gain-low intake animals compared to the other groups. Aldosterone is secreted by the adrenal gland and plays a critical role in electrolyte and fluid homeostasis, which are regulators of blood pressure. While we cannot explain what function this pathway may have in the spleen as a component of feed intake and gain traits in cattle, it is of interest that genes within this pathway were also identified as over-represented in a recent GWAS for feed conversion ratio in cattle (Santana et al., 2014).

In summary, we have detected DEG and pathways within the spleen that appear to be associated with an animal's feed intake and gain phenotype. While it may not be surprising that immune response genes were identified within the spleen due to its function as a lymphoid organ, it may be important to note that MHC class II genes were also identified as differentially expressed in the rumen and small intestine of different animals with feed and intake phenotypes. Another class of genes, involved in stress response, was also identified in the small intestine (Lindholm-Perry et al., 2016) indicating that these functions may represent a contribution to these phenotypes by several organs. The genes involved in lipid metabolism and the potential role of the GBP genes as CNV appear to be unique to the spleen. It will be of interest to evaluate these genes in other populations of cattle with similar phenotypes to confirm whether they maintain an association with feed intake or gain.

## Author note

Mention of trade name proprietary product or specified equipment does not constitute a guarantee or warranty by the USDA and does not imply approval to the exclusion of other products that may be suitable. USDA is an equal opportunity provider and employer.

## Author contributions

AL Designed experiments, analyzed data, and drafted the manuscript; RK Performed tissue collections, microarray experiments, microarray QC; BK, WS Conceived and designed CNV analyses; LK Performed gene differential expression analysis; HF Performed animal phenotyping and analyses for animal selection, collected samples. All authors made significant contributions drafting and editing the manuscript.

### Conflict of interest statement

The authors declare that the research was conducted in the absence of any commercial or financial relationships that could be construed as a potential conflict of interest. The reviewer FL and handling Editor declared their shared affiliation, and the handling Editor states that the process nevertheless met the standards of a fair and objective review.
